# Simple mechanics of protein machines

**DOI:** 10.1098/rsif.2019.0244

**Published:** 2019-06-19

**Authors:** Holger Flechsig, Alexander S. Mikhailov

**Affiliations:** 1 Nano Life Science Institute (WPI-NanoLSI), Kanazawa University, Kakuma-machi, 920-1192 Kanazawa, Japan; 2 Department of Physical Chemistry, Fritz Haber Institute of the Max Planck Society, Faradayweg 4-6, 14195 Berlin, Germany

**Keywords:** complex systems, self-organization, elastic networks, mechanochemical motions in enzymes, molecular motors, allosteric effects

## Abstract

While belonging to the nanoscale, protein machines are so complex that tracing even a small fraction of their cycle requires weeks of calculations on supercomputers. Surprisingly, many aspects of their operation can be however already reproduced by using very simple mechanical models of elastic networks. The analysis suggests that, similar to other self-organized complex systems, functional collective dynamics in such proteins is effectively reduced to a low-dimensional attractive manifold.

## 1. Introduction

To a large extent, the living cell is a population of interacting molecular machines [1]. These protein machines, acting as motors and pumps or performing operations with other biomolecules, such as DNA, underlie basic functions of the cell. Understanding of their mechanisms is essential for molecular biology and for prospective biotechnology applications. Single-molecule experiments could provide much information on the dynamics of protein machines [2–5]. More recently, high-speed atomic force microscopy methods have allowed direct visualization of conformational motions at nanoscale resolution in real time [6].

Molecular structures and equilibrium conformations of almost all proteins are known. They are determined through a combination of X-ray diffraction experiments, cryo-electronic microscopy and other techniques, and can be found in the Protein Data Bank (PDB). Moreover, conformational dynamics of proteins is sufficiently well reproduced in all-atom molecular dynamics (MD) simulations. Hence, it may seem that just some special MD simulations have to be performed in order to unveil the operation mechanisms of protein machines. In practice, severe difficulties are encountered if one attempts to do this.

All-atom MD simulations of proteins are extremely demanding in terms of the computation time. Even with the most powerful supercomputers, the dynamics of a protein can be traced only up to microsecond times. The best achievement so far has been that, by employing special hardware and for a very small protein, a trajectory of a millisecond duration could be obtained [7]. This is frustrating because operation cycles of protein machines usually take tens of milliseconds. Thus, even a single cycle for such a machine could not have been followed in MD simulations and this would probably also not be done in the near future. It is astounding that such a high degree of complexity, comparable to what is characteristic for the global climate forecast or for modelling of big social systems, is found already at the nanoscale, for macromolecules with only tens of thousands of atoms.

Within the last century, a substantial progress in understanding large complex systems has been made (e.g. [8]). It is known that, in order to be functional, i.e. to have robust and predictable dynamics, such systems should possess special organization. They need to be organized into a hierarchy of dynamical subsystems, each hierarchical level with its own separate time scale [9]. Moreover, it is only rarely possible to deduce the descriptions at a higher level from the dynamics at the lower level. Instead, phenomenological models based on collective variables, or ‘order parameters’, are usually employed [10].

Furthermore, two kinds of mathematical models for complex systems can be distinguished. The ‘realistic’ models are used to provide accurate quantitative predictions for a particular considered case (as, for example, in a weather forecast). Typically, they would include many variables and parameters, and their results still need to be interpreted and understood. On the other hand, ‘simple’ mathematical descriptions for complex systems are also broadly employed. The intention of such reduced models is not to yield accurate quantitative predictions, but rather to help in understanding of the principal mechanisms involved (see also [11]).

Good examples of both approaches are provided by brain research. There exist realistic models for chemical and electrical processes within a neural cell. However, they are complicated and therefore used only for single neurons or small populations of them. Large-scale modelling is instead performed by means of greatly simplified models of neural networks. In such networks, a neuron can even be treated as an automaton with just a few states. Thus, actual physical and chemical processes are not resolved. Nonetheless, simple neural network models play a fundamental role in understanding of the brain.

Simple models of complex systems represent investigation vehicles rather than computational tools. Such models are often built by stressing one aspect of functional behaviour and not resolving the rest. In this manner, one can better see what interactions between the elements are responsible for a specific function. It should be noted that such approach forms the basis of *constructive* biology aimed at principal understanding of how various biological functions emerge [12].

For proteins, both ‘realistic’ and ‘simple’ models are being used, although the distinction is often not clearly made. Their dynamics can be resolved either at the level of atoms, or at the level of groups of atoms (such as residues), or at that of entire protein domains. These three levels possess different characteristic time scales, from picoseconds for single atoms, to nanoseconds for atomic group residues, and to microseconds or even milliseconds for motions of the domains. The models at the last two levels are called coarse-grained.

The coarse-grained descriptions are typically judged by how precise they are. With this perspective, an impressive progress has been made and fairly accurate, but still computationally fast formulations exist. However, the increasingly ‘realistic’ coarse-grained descriptions get loaded with details and become less transparent in comparison to the original physical models that are quite simple.

Our focus in this review is not on how accurate the coarse-grained descriptions for protein dynamics could be. Instead, we concentrate on the simplest mechanical models of protein machines and want to demonstrate how much can be already learned while exploring them.

## 2. Ligand-induced mechanochemical motions in enzymes

Protein machines represent a special class of enzymes. An enzyme is a protein that acts as a single-molecule catalyst accelerating a chemical reaction. The reaction event itself takes place at an active centre inside the enzyme. Catalytic reactions can involve conversion of one or two substrates into one or two products, and several intermediate products can also be formed. For simplicity, we shall however assume below that one substrate molecule is converted into one product molecule in a turnover cycle.

Hence, the considered reaction has three steps: (i) a substrate binds to the protein at its active centre and forms a substrate–enzyme complex ES, (ii) within the complex, the substrate is converted into a product and a product–enzyme complex EP is formed, (iii) the product is released and the enzyme returns to its free form E, i.e.

2.1 E+S⇌ ES ⇌ EP ⇌E+P.

The steps are generally reversible, so that an enzyme can also operate in the opposite direction, even though the probability of reverse cycles might be small.

A protein could have just provided a static support and an appropriate environment for an active centre where a catalytic event takes place. In most enzymes, binding or release of a ligand (i.e. of a substrate or a product) and transitions in the ligand state are however accompanied by conformational changes, so that ligand-induced mechanochemical motions arise.

The origin of such motions can be easily understood: when a ligand binds to a protein, a new mechanical object, i.e. the ligand–protein complex, is formed. Generally, this new object includes additional interactions and, therefore, it has a different equilibrium state. Thus, after ligand binding, a process of conformational relaxation from the original equilibrium state of a free protein to the new equilibrium state of the complex should take place. Similar internal mechanical motions can be triggered by other transitions within a turnover cycle. The database [13] contains information on ligand-induced conformational changes in many proteins.

**Figure 1.**
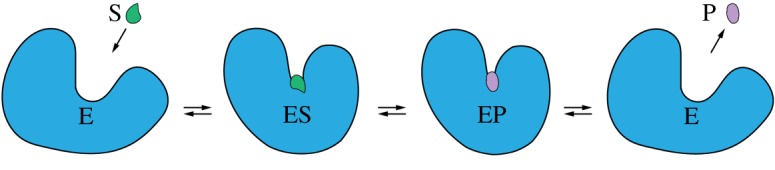
A sketch of mechanochemical motions accompanying the catalytic turnover cycle of an enzyme. (Online version in colour.)

Hence, every transition in the reaction (2.1) is generally accompanied by a change in the shape of an enzyme, i.e. by some *mechanochemical motion* (figure 1). At the end, the enzyme returns to its original free state and, thus, to its original equilibrium conformation. This is repeated in each next cycle. As a result, the enzyme effectively behaves like an oscillator, repeatedly changing its shape.

Ligand-induced mechanochemical motions in enzymes are *functional*. Their roles can be, for example, to create an optimal configuration for the catalytic conversion event, to transport ligand(s) to an active centre and to open the gates for product(s) release. However, mechanochemical motions can be also employed to produce forces and to manipulate other macromolecules. In such cases, an enzyme operates as a nano-machine.

Since the role of catalytic chemical reactions in protein machines is just to power the mechanical activity, many of them use the same reaction of ATP hydrolysis. In this reaction, a molecule of ATP binds to a protein and becomes converted in its active centre to the products, ADP and phosphate (Pi), that are afterwards released. Through each hydrolysis event, an energy of about 20*k*_B_*T* becomes supplied. Because the difference in energies is high, this reaction is practically irreversible.^1^

## 3. Active dimer model of an enzyme

Protein machines often have a domain structure, i.e. they consist of two or more domains connected by flexible joints. Ligand-induced mechanochemical motions in such proteins represent relative translational or rotational movements of the domains. Although the details of the domain structure and of the dynamics obviously depend on a particular protein, the basic mechanism can be well illustrated by a simple model of an *active dimer* [14,15].

**Figure 2.**
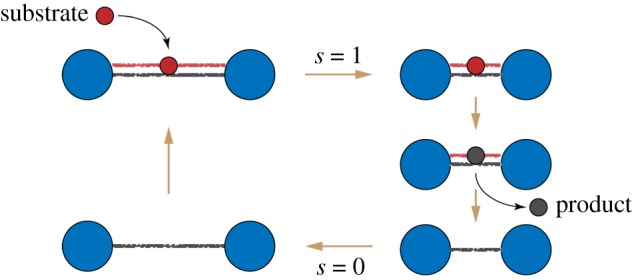
The cycle of an active dimer. A substrate (red) binds and induces shortening of the dimer. Then, the substrate is converted to a product (black), and the product is released. Finally, the dimer returns to its original shape. (Online version in colour.)

This dimer has two beads that correspond, as an idealization, to two protein domains (figure 2). The beads are connected by an elastic link with natural length *l*_0_ and stiffness constant *k*_0_. When a substrate arrives, it introduces an additional elastic link between the two domains, with short natural length *l*_*c*_ and stiffness *k*_*c*_. Thus, substrate binding induces a mechanochemical motion, i.e. shortening of the distance between the beads. When the new equilibrium configuration is however reached, a reaction converting the substrate into a product takes place and, as is for simplicity assumed, the product is immediately released. As a result, the additional link connecting the two domains disappears and, by a reverse relaxation process, i.e. another mechanochemical motion, the dimer returns to its original equilibrium configuration. With the arrival of a further substrate, the cycle is repeated again. Reverse transitions can be allowed, but we shall consider a model without them.

Since the product is instantaneously released once it has been formed, the dimer can be found in only two states: *s* = 0 (the free dimer) and *s* = 1 (the dimer with a bound ligand, i.e. with a substrate). Elastic energies in these two states are

**Figure 3.**
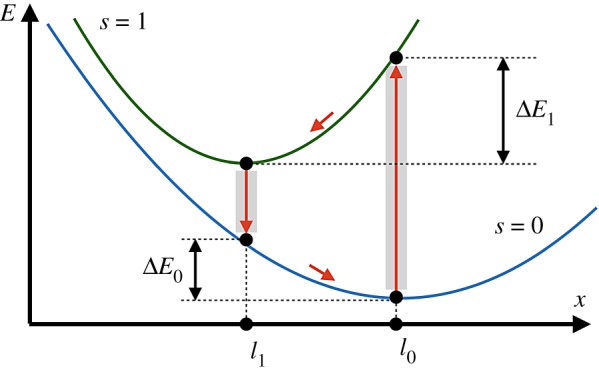
Energy diagram of the active dimer. Two branches of the dependence of elastic energy *E* on distance *x* between the beads for configurations with (*s* = 1) and without (*s* = 0) a ligand are shown. Transitions between the branches occur at *x* = *l*_0_ and *x* = *l*_1_; they are followed by relaxation to new equilibrium states. Within each turnover cycle, energy Δ*E*_0_ + Δ*E*_1_ is dissipated in mechanical motions and the same energy is externally supplied through the ligand. (Online version in colour.)

3.1 $$E_{s}(x)=\frac{1}{2}k_{0}{\left(x-l_{0}\right)}^{2}+\frac{1}{2}sk_{c}{\left(x-l_{c}\right)}^{2},$$

where *x* is the distance between the beads. The two energy branches are shown in figure 3. The equilibrium states of the dimer are *x* = *l*_0_ for *s* = 0 and *x* = *l*_1_ for *s* = 1 where *l*_1_ = (*k*_0_*l*_0_ + *k*_*c*_*l*_*c*_)/(*k*_0_ + *k*_*c*_). Note that, effectively, the two beads are connected in the state *s* = 1 by an elastic link with stiffness *k*_1_ = *k*_0_ + *k*_*c*_ and natural length *l*_1_.

The dimer is immersed into viscous fluid and, as characteristic for micro- and millisecond time scales, inertia is absent and its internal motions are overdamped. Its dynamics is described by the Langevin equation

3.2 $$\frac{\text{d}x}{\text{d}t}=-\Gamma \frac{\partial E_{s}}{\partial x}+\xi (t),$$

where *Γ* is the mobility of a bead and *ξ*(*t*) represents thermal noise with the correlation function

3.3 $$\langle \xi (t)\xi (t^{\prime })\rangle =2\Gamma k_{B}T\delta (t-t^{\prime }),$$

where *T* is the temperature and *k*_B_ is the Boltzmann constant.

Stochastic transitions between two energy branches, that correspond to binding of a substrate and product release, are assumed to take place only near the equilibrium conformations. Their rates are *w*_01_ = *c*_*s*_*ν* (for substrate binding) and *w*_10_ (for product release). Note that the rate of substrate binding is proportional to substrate concentration *c*_*s*_.

Probability distributions *p*_0_(*x*) and *p*_1_(*x*) to find the dimer in states *s* = 0, 1 with distance *x* between the beads obey a system of two coupled Fokker–Planck equations

3.4 $$\begin{array}{rl} \frac{\partial p_{0}}{\partial t} & =\frac{\partial }{\partial x}\left(\Gamma \frac{\partial E_{0}}{\partial x}p_{0}\right)+\Gamma \;k_{B}T\frac{\partial^{2}p_{0}}{\partial x^{2}}\\ & \;-w_{01}\delta (x-l_{0})p_{0}(x)+w_{10}\delta (x-l_{1})p_{1}(x) \end{array}$$

and

3.5 $$\begin{array}{rl} \frac{\partial p_{1}}{\partial t} & =\frac{\partial }{\partial x}(\Gamma \frac{\partial E_{1}}{\partial x}p_{1})+\Gamma \;k_{B}T\frac{\partial^{2}p_{1}}{\partial x^{2}}\\ & \;-w_{10}\delta (x-l_{1})p_{1}(x)+w_{01}\delta (x-l_{0})p_{0}(x) \end{array}.$$

Such dimer behaves as a stochastic oscillator that alternates between the two states, with conformational relaxation following each transition between them.

The persistent oscillations are powered by the energy supplied with substrates. When a substrate binds, it brings the energy *ε*_*s*_ = *E*_1_(*x* = *l*_0_) − *E*_0_(*x* = *l*_0_). On the other hand, when a product is released, it removes the energy *ε*_*p*_ = *E*_1_(*x* = *l*_1_) − *E*_0_(*x* = *l*_1_). Therefore, the energy provided to the dimer in each cycle is Δ*E* = *ε*_*s*_ − *ε*_*p*_ = (1/2)(*k*_0_ + *k*_1_)(*l*_0_ − *l*_1_)^2^ where *k*_1_ = *k*_0_ + *k*_*c*_. It can be checked that it is equal, as should have been expected, to the sum of the energies Δ*E*_1_ and Δ*E*_0_ dissipated in the mechanochemical motions.

When conditions Δ*E*_1_ ≫ *k*_B_*T* and Δ*E*_0_ ≫ *k*_B_*T* are satisfied, thermal fluctuations do not significantly affect the dynamics. The oscillation period is determined by the times of mechanochemical relaxational motions and the waiting times are determined by transition rates *w*_10_ and *w*_01_.

The active dimer represents an idealization of an enzyme with mechanochemical motions inside its turnover cycle. As shown in the next section, it can be converted to a molecular motor by using different ratchet mechanisms.

## 4. Ratchet translocation mechanisms

In classical engineering, mechanical ratchets are commonly employed to transform oscillations into steady translational or rotational motions. It is by a ratchet that reciprocal spring length oscillations in a clock are transformed into the rotational motion of its hands. Hence, it should not be surprising that similar techniques are also broadly used by molecular motors at the nanoscale.

**Figure 4.**
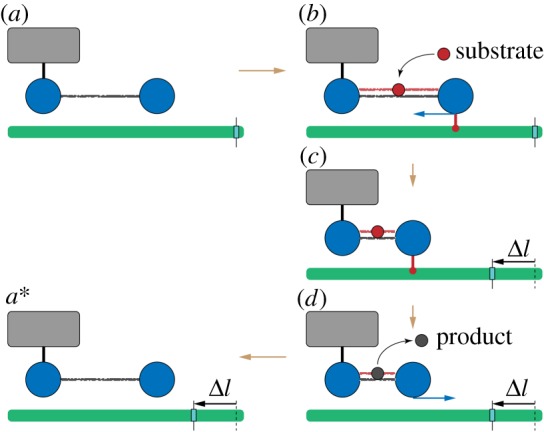
A ratchet motor. The left bead of the dimer is immobilized (schematically shown by a link to the grey box). The filament (green) is mobile and can slide. (*a*) Initially, the dimer is in the expanded state without a ligand. (*b*) When a substrate arrives, the right bead of the dimer forms a connection to the filament and holds it. (*c*) As the dimer contracts, it moves the sliding filament to the left. (*d*) Once the product is formed and immediately released, the connection between the dimer and the filament disappears, and the dimer freely expands. (a*) After one machine cycle, the dimer returns to its initial configuration, but the filament becomes displaced to the left by the distance Δ*l* = *l*_0_ − *l*_1_. To visualize the displacement, a mark is attached to the filament. See also the electronic supplementary material, video S1. (Online version in colour.)

The most straightforward way by which the active dimer can be converted into a motor is illustrated in figure 4. In this case, the function of the motor is to steadily move (i.e. translocate) a filament. This function is implemented by means of the classical ratchet mechanism. The dimer is immobilized by fixing the left domain to a solid support, whereas the right domain is free. In the first half of the cycle, the mobile right domain holds the filament and moves it. In the second half, however, the connection to the filament is absent and the domain moves back without it. As easily seen, in each cycle the filament becomes translocated in the left direction by distance Δ*l* = *l*_0_ − *l*_1_.

This ratchet mechanism cannot however be readily implemented at the nanoscale. Indeed, it requires precise localization and fixation of the motor with respect to the filament—but strong thermal fluctuations may prevent this.^2^

**Figure 5.**
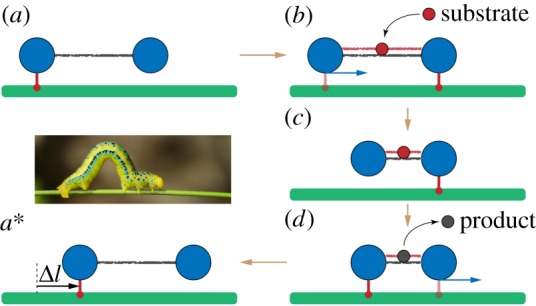
An inchworm translocation motor. The filament (green) is immobile and the dimer machine can actively translocate itself along it. (*a*) Initially, the left bead of the dimer is connected to the filament and holds it. (*b*) When a substrate arrives, a connection between the right bead and the filament is established and then the left bead gets disconnected. (*c*) The dimer contracts, bringing the left bead closer to the immobile right bead. (*d*) When a product is formed and instantaneously released, the left bead re-establishes a connection to the filament and becomes immobile, whereas the right bead is disconnected. (a*) The free dimer expands and reaches its initial conformation. After one cycle, its location on the filament is shifted by the distance Δ*l* = *l*_0_ − *l*_1_. The inset shows an animal inchworm. See also the electronic supplementary material, video S2. (Online version in colour.)

Such limitation is absent in the *inchworm* translocation mechanism (figure 5). Here, the filament is always held by at least one domain. When the right domain is connected to the filament, the dimer contracts and the left domain moves to the right. After that, the left domain establishes a connection to the filament and, at the same time, the right domain becomes free. Now, as the dimer expands, the right domain moves forward. As a result, in each cycle the centre of mass of the motor is shifted by Δ*l* = *l*_0_ − *l*_1_.

While in the active dimer model all energy supplied with a ligand is dissipated in internal mechanochemical motions within the turnover cycle, a fraction of this energy is used to produce external mechanical work in a motor (e.g. to transport a filament through viscous fluid or to translocate the motor along an immobile filament).

There are also other possibilities to convert cyclic shape changes into a steady progressive motion. For example, the molecular dimer motor myosin V is ‘walking’ over an actin filament, repeatedly lifting and moving forward one of its heads. Remarkably, this walking myosin motion could be directly visualized by using high-speed atomic force microscopy [16].

## 5. Elastic network models of proteins

The elementary units of proteins are residues (amino acids) and therefore it is natural to work with the coarse-grained descriptions at the level of such atomic groups. There are 20 essential amino acids and they are arranged into a long polypeptide chain. Because of the interactions between the residues, such polymer chains fold into unique equilibrium conformations that define the protein native form.

In a compactly folded conformation, each residue has several other residues in its neighbourhood and it effectively interacts only with them. Under relatively weak local deformations of the folded state, the pattern of contacts between the residues, or the contact map, remains preserved and only distances between neighbour residues are changed. Such deformations can therefore be viewed as *elastic*, in contrast to plastic deformations that would have involved partial unfolding or refolding, and thus a change in the contact map.

This observation leads to a phenomenological description of a protein in terms of an elastic network, with individual residues represented by point-like particles and elastic potential interactions between them. Essentially, the network represents a set of beads connected by elastic springs. Such simple description was first proposed (but at the atomistic level) by Tirion in 1996 [17]. Below, we shall use a variant of the description that was formulated by Bahar *et al*. [18] (see also [19]).

The energy of a network of *N* beads *i* = 1, 2, …, *N* connected by a pattern of elastic springs with (identical) stiffness constants *k* is

5.1 $$E=\frac{k}{2}\sum_{i<j}A_{ij}(d_{ij}-d_{ij}^{\left(0\right)}{)}^{2},$$

where *d*_*ij*_ = |**R**_*i*_ − **R**_*j*_| is the length of a spring that connects beads *i* and *j* at positions **R**_*i*_ and **R**_*j*_ and $d_{ij}^{\left(0\right)}$ is the natural length of this spring; matrix *A*_*ij*_ with elements 0 or 1 defines the pattern of connections between the beads.

To obtain an elastic network for a protein, its experimentally known equilibrium conformation from the PDB is used. The equilibrium position $R_{i}^{\left(0\right)}$ for every residue *i* is determined by the coordinates of the *α*-carbon atom of this residue in the equilibrium PDB state. Then, equilibrium distances $d_{ij}^{\left(0\right)}=|R_{i}^{\left(0\right)}-R_{\;j}^{\left(0\right)}|$ are computed for all pairs (*i*, *j*) of residues in the protein. If, for a given pair, the equilibrium distance is shorter than a cutoff length *l*_cut_, the respective two beads are made connected by an elastic spring. Hence, the connection matrix is chosen as *A*_*ij*_ = 1 if $d_{ij}^{\left(0\right)}<l_{\mathrm{cut}}$ and *A*_*ij*_ = 0 otherwise. Furthermore, the natural lengths of the springs are made equal to the equilibrium PDB distances $d_{ij}^{\left(0\right)}$ between them. Thus, an elastic network becomes constructed [18,19] whose equilibrium state coincides with the known equilibrium PDB conformation of the considered protein.

Note that, while being quadratic in terms of distance changes between the beads, the elastic energy (5.1) is a more complex function of coordinates **R**_*i*_. Generally, an elastic network is therefore a *nonlinear* mechanical system.

Often, additional linearization in terms of the deviations $r_{i}=R_{i}-R_{i}^{\left(0\right)}$ from the equilibrium positions of residues is performed, leading to a set of normal modes on which the subsequent linear analysis relies. Thus, an approximate expression for the elastic energy, quadratic in variables **r**_*i*_, is instead employed. It has been pointed out in [20] that even nonlinear conformational dynamics of proteins can be however investigated by using the elastic energy (5.1).

At the time scales exceeding picoseconds, inertial effects are negligible and the dynamics is overdamped. Therefore, neglecting hydrodynamic effects, the equations of motion for the beads corresponding to protein residues are (for *i* = 1, 2, …, *N*)

5.2 $$\frac{dR_{i}}{dt}=-\gamma \frac{\partial E}{\partial R_{i}}+f_{i}(t),$$

where *γ* is the mobility of the beads (for simplicity, assumed to be the same for all of them). These Langevin equations include independent thermal noises with components *f*_*α*,*i*_(*t*) for *α* = *x*, *y*, *z* that have correlation functions

5.3 $$\langle f_{\alpha ,i}(t)f_{\beta ,j}(t^{\prime })\rangle =2\gamma k_{B}T\delta_{\alpha \beta }\delta_{ij}\delta (t-t^{\prime }).$$

Strong simplifications are obviously involved in this model and therefore the questions can be asked: Would not it be better to assume that the stiffness constant *k* for a spring depends on what pairs of residues are connected by it? Should not it perhaps also depend on the natural length, so that the longer springs are more soft? Should not the mobility *γ* of the residues depend on their positions within a protein and perhaps be higher on the surface of it?

Paying attention to such questions, different variants of elastic network models for proteins have been proposed and are employed (for comparison and discussion, see [21]). Moreover, an iterative learning algorithm based on experimental nuclear magnetic resonance (NMR) data for a large set of proteins could be used to determine optimal residue-specific stiffness constants that were also dependent on the natural length [21].

The intrinsic difficulty of elastic network models is that they do not allow partial unfolding and refolding during the dynamics of a protein. The pattern of spring connections is determined by the equilibrium conformation of a protein and remains fixed. To some extent, such processes can be taken into account by modifying interaction potentials between the beads [22–25].

It should be noted that there are also other structure-based descriptions for proteins, where unfolding and refolding may take place and where more complex and diverse interactions between the residues are assumed. For a survey of such coarse-grained models, see [26,27].

Our focus in this review is however on simple mechanical descriptions. Therefore, we shall rely on the original elastic network model as formulated above.

## 6. Conformational dynamics in protein machines

Ligand-induced mechanochemical motions that play a principal role in the operation of protein machines are conformational relaxation processes in such macromolecules. At the level of domains, they could be already reproduced in the active dimer model considered above. The residue-level elastic network descriptions are much more complex and conformational relaxation processes in such models should be thoroughly explored.

**Figure 6.**
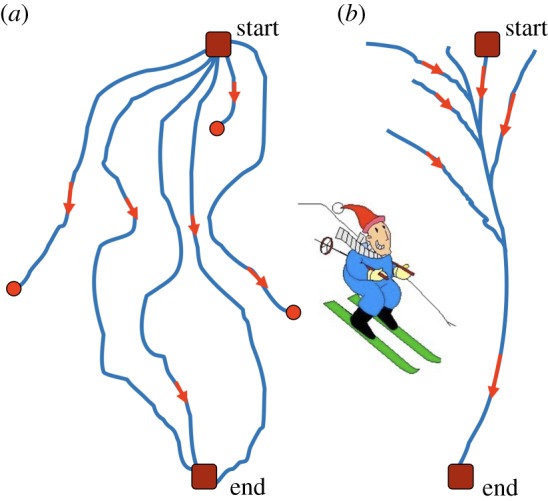
A sketch of downhill skiing over (*a*) a ragged slope and (*b*) a slope with a narrow valley. (Online version in colour.)

In the absence of thermal noises, the dynamics of an elastic network represents its relaxation to a state with the minimal elastic energy (5.1). Generally, the energy landscape can be quite complicated and include many additional minima, in addition to the equilibrium reference state (*E* = 0). In a ragged energy landscape, relaxation would be typically terminated in one of such metastable states (figure 6*a*).

**Figure 7.**
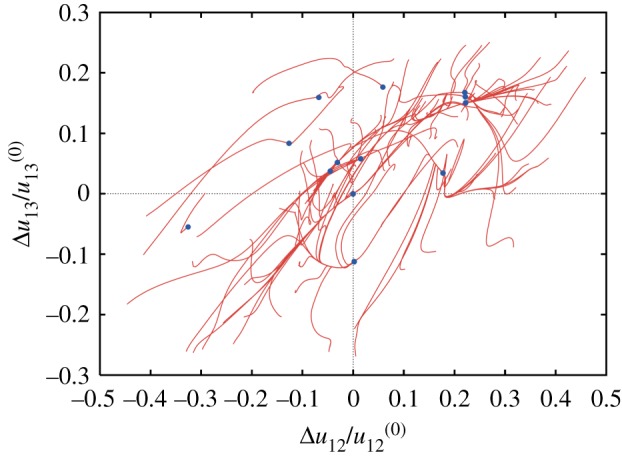
Conformational relaxation in a random elastic network. Each of the 100 displayed relaxation trajectories starts from a different initial conformation; blue dots indicate final states. Projection on the plane of normalized deviations of distances between three randomly chosen labels from their respective values in the equilibrium reference state. The reference state corresponds therefore to the origin of coordinates. Adapted from [20]. (Online version in colour.)

When elastic networks are *randomly* constructed, they indeed tend to have ragged energy landscapes and thus resemble glass systems [20]. Figure 7 shows the pattern of relaxation trajectories in a typical random network. As we see, only a few of the 100 displayed trajectories ended in the true equilibrium state. All other trajectories terminated in various metastable states.

**Figure 8.**
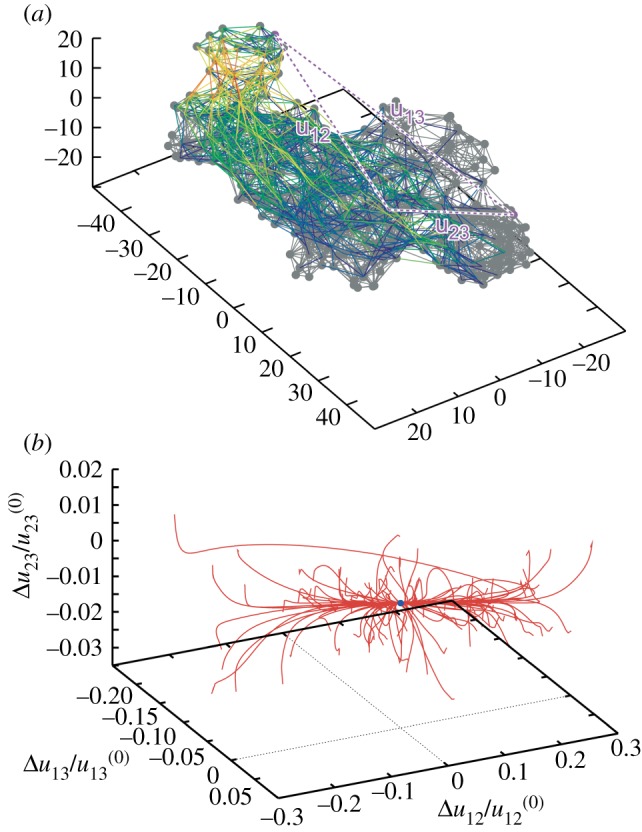
Elastic network of a single *β*-subunit of the molecular motor F1-ATPase (*a*) and the set of relaxation trajectories for this network (*b*). Links in the elastic network are coloured according to their deformations in the slowest normal mode (with red for the most strong and blue for the most weak deformations). Each of the 100 trajectories starts from a different initial network conformation. Trajectories are displayed in the space of relative distance changes between labels (1,2,3) indicated in the elastic network. The equilibrium state corresponds to the origin of coordinates. Adapted from [20]. (Online version in colour.)

A completely different relaxation pattern is characteristic for protein machines [20,28]. Figure 8 shows the pattern of conformational relaxation in a single *β*-subunit of the rotary molecular motor F1-ATPase (it consists of three *β*- and three *α*-subunits forming a ring). The metastable states are absent and all trajectories return to the equilibrium reference conformation, even though the distance *u*_12_ between labels 1 and 2 could have changed by up to 30%.

Remarkably, another important feature can be noticed in figure 8. Starting from various initial conditions, the trajectories converge to a narrow bundle that leads to the equilibrium state. This suggests a special *funnel* organization of the elastic energy landscape: this landscape includes a narrow valley with steep walls that leads to the equilibrium state. The motions starting at different positions first fall into this valley and then continue along the bottom of it (figure 6*b*).

**Figure 9.**
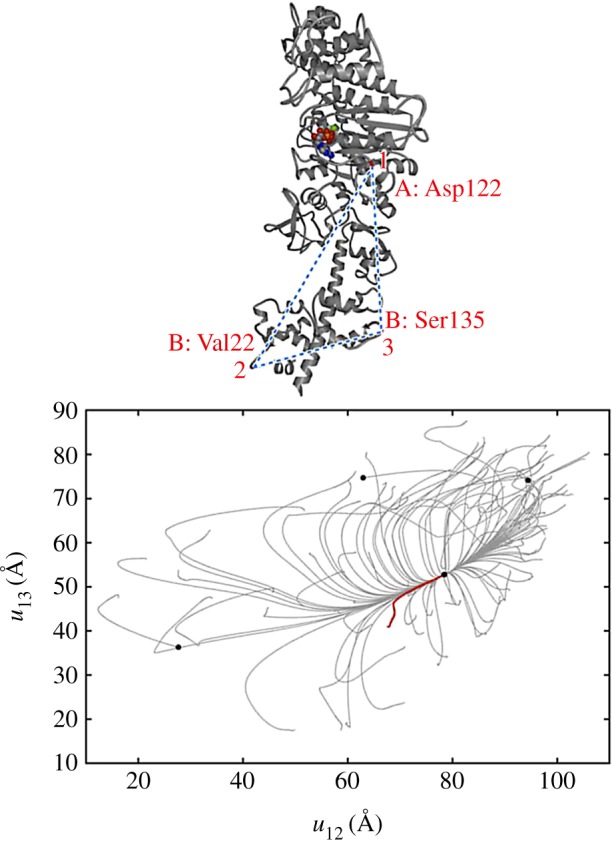
Conformational relaxation in myosin V. Here, 100 relaxation trajectories starting from different initial network conformations are plotted in the plane of distances between the labels (1,2,3) indicated above. Black dots mark the equilibrium and the metastable states reached. The ATP-bound equilibrium structure is taken as a reference state. The red trajectory corresponds to the conformational transition upon ATP binding. Adapted from [28]. (Online version in colour.)

Similar behaviour could be found when conformational relaxation in the elastic networks of other motor proteins, such as muscle myosin [20], myosin V (figure 9) and kinesin KIF1A [28], hepatitis C virus (HCV) helicase [29], various membrane ABC transporters [30] and several superfamily 2 helicases [31], was examined. This suggests that it may represent *a common property* of the proteins operating as motors or machines.

The funnel energy landscape is known to be characteristic for protein folding. It ensures that, at its last stage, the folding proceeds along a definite pathway and leads to a unique native conformation of a protein [32]. By contrast, the landscape with the energy (5.1) is for elastic deformations of a protein already in its folded state. Nonetheless, the funnel structure of this landscape should have a similar interpretation: it ensures that the protein has definite conformational motions, all proceeding close to the same pathway.

In a macroscopic mechanical device operating as a machine, well-defined movements of its parts are repeated in each cycle. The analogous movements in protein machines represent mechanochemical motions in a protein. The special structure of the energy surface, with a narrow valley and steep walls, allows a molecular device to operate similar to a classical machine. In this way, transverse fluctuations become suppressed and the motion becomes effectively low-dimensional, i.e. characterized by one or a few collective mechanical coordinates.

The analysis shows that functional conformational motions in machine proteins are typically slow [20]. When additional linearization of dynamical equations is performed and the normal modes are determined, they correspond to the modes with low relaxation rates. Hence, their existence implies that a gap is present in the relaxation rate spectrum of the normal modes of a protein [20].

Reduction of collective dynamics to a low-dimensional attractive manifold is typical for functional complex systems and represents a characteristic *self-organization* effect. When a similar reduction is found for proteins, their relaxation dynamics can therefore be described as being self-organized.

According to the general theory of complex systems [10], the amplitudes corresponding to slow collective motions in proteins can be viewed as *order parameters* that enslave other, fast conformational motions and control them. Note that a connection to the domain-level descriptions becomes hence established: such order parameters may yield natural variables by which relative motions of the domains can be described.

## 7. Protein evolution and design of machines

Ordered slow collective motions in proteins could have emerged in the process of biological evolution. During this process, proteins should become optimized for their machine functions—and ordered mechanochemical motions are a pre-requisite for that. Currently, the emergence and evolution of protein machines is a research subject of much interest.

While similar investigations for actual proteins remain to be performed, the question has been addressed by looking at whether it would be possible to construct an elastic network with ordered collective motions by running a computer evolution [20] (see also [33,34]). It has been demonstrated that, as a result of such an evolution, elastic networks with the properties strongly resembling those of actual protein machines can be indeed designed [20].

**Figure 10.**
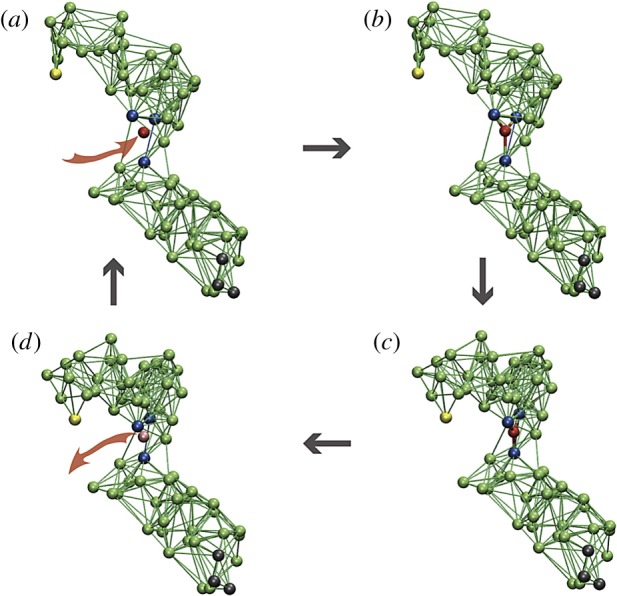
The cycle of a model elastic network machine [20]. (*a*) Initially, the machine is in its equilibrium state. (*b*) A substrate (red) establishes elastic links to three beads (blue) in the hinge region between the two domains. (*c*) The machine changes its conformation to the new equilibrium state. (*d*) In this state, a reaction converting the substrate into product takes place and the product is instantaneously released. After that, the machine returns to its original conformation, completing the cycle. When the machine is used to construct a motor, three black beads in the lower domain are immobilized and the yellow bead in the upper domain interacts with the filament. Reproduced from [35]. See also the electronic supplementary material, video S3. (Online version in colour.)

The constructed network consists of two stiff domains connected by a flexible hinge (figure 10). The pattern of connections in the hinge region is optimized to ensure ordered conformational motions described by a single collective mechanical coordinate. These collective motions are slow and separated by a gap from other conformational motions in the network. Moreover, there are no metastable states in the neighbourhood of them. Therefore, after any perturbation, the designed network moves back to its equilibrium conformation along a unique pathway.

By using the constructed elastic network, a model protein machine could be furthermore designed [20]. Binding of a substrate (figure 10) leads to a conformational change from the open to a closed equilibrium conformation. This mechanochemical motion is also slow and proceeds along a well-defined pathway. In the closed state, the substrate is converted into a product and the product is released. Then, the backward conformational motion to the original equilibrium state takes place. Thermal fluctuations could be included into the model [20], revealing that mechanochemical motions are robust with respect to them. A detailed description of the model machine is given in [35].

This designed machine operates cyclically, similar to an oscillator, and its operation is powered by the energy supplied with the substrates. In fact, it represents a structurally resolved version of the active dimer in §3.

In the original version, Langevin stochastic dynamics was employed [20] and therefore hydrodynamic effects could not be resolved. Hydrodynamic flows could be however later taken into account [36]. This was done by immersing the designed machine into an environment consisting of solvent particles whose interactions were characterized by multi-particle collision dynamics [37]. Repulsive or attractive forces between the machine beads and solvent particles, corresponding to hydrophobic or hydrophilic interactions, could be introduced. Simulations have shown that, even under hydrodynamic fluctuations, ordered collective motions of the machine persist [36]. Remarkably, they proceed along the same pathway as in the Langevin dynamics, although the motion along this pathway becomes modified.

**Figure 11.**
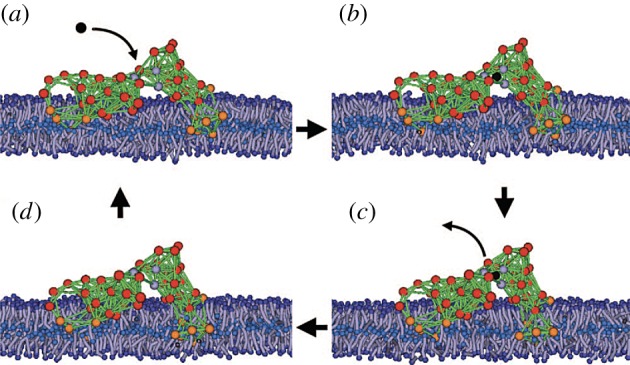
The model protein machine in a biological membrane. The lipids are modelled as short polymer strings. Orange beads are hydrophobic and red beads are hydrophilic. The solvent is included into the simulations, but its particles are not displayed. Adapted from [38]. See also the electronic supplementary material, video S4. (Online version in colour.)

The designed machine has also been used in a model study of active inclusions in biological membranes [38]. The membranes were formed by lipids, modelled as short polymer strings and immersed into the solvent with the multi-particle collision dynamics. Two groups of machine beads in both its domains were made hydrophobic, so that they preferred to stay inside the membrane (figure 11). Lipid flows accompanying cyclic contractions of this machine, corresponding to an active protein inclusion in a biomembrane, have been determined and analysed [38].

By using the designed machine, a model molecular motor could be constructed [35] by using the ratchet mechanism shown in figure 4.

**Figure 12.**
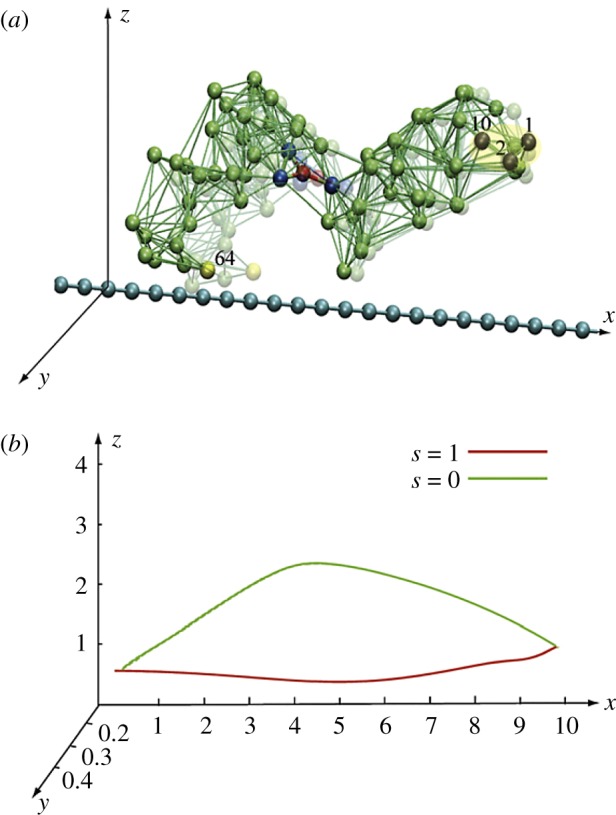
Construction of a model ratchet motor. (*a*) Beads 1, 2 and 10 are fixed, immobilizing one domain. Bead 64 can interact with the force centres (blue beads) on the filament. The filament can only slide along its direction. (*b*) The trajectory of bead 64 within one cycle. In the ligand-bound state *s* = 1, the bead comes close to the filament, establishes interactions, and moves it. In the second half of the cycle (*s* = 0), this bead is separated from the filament and moves back without holding it. Thus, the filament becomes progressively translocated after each cycle. Adapted from [38]. See also the electronic supplementary material, video S5. (Online version in colour.)

The interactions with the filament were then resolved. Three beads in one motor domain were immobilized, thus fixing the position of this domain (figure 12*a*). The second domain performed swinging motions, repeated in each cycle. They could be traced by monitoring positions of the end bead (figure 12*b*). Approximately, the bead moved along a straight line after binding of a substrate. The filament was positioned close to this line and it could only slide (with viscous friction) along its direction. Force centres were placed at regular intervals along it.

The end bead had short-ranged attractive potential interactions with the force centres. Hence, in one part of the cycle the mobile domain moved to the right while grasping the filament, whereas it moved back in the second part of the cycle without it. Thermal fluctuations for the beads and the filament were further included and an external force (i.e. the load) could be introduced.

Remarkably, already this simple model allowed the analysis of the stepping behaviour in the weak and strong coupling regimes, yielding statistics [35] similar to that in real protein motors (see also [34]). The stall effect could also be reproduced.

## 8. Operation cycles of molecular motor hepatitis C virus helicase

As a result of natural biological evolution, protein motors employ various strategies to transform internal mechanochemical motions into the directional transport. They are well adapted to different functions they should execute. Motors that have to transport cargo through the cell, such as the cytoskeletal motors kinesin and dynein or the myosin V motor, are equipped with two legs whose coordinated movements allow them to walk along the tracks without modifying them [39,40]. Other motors may have the task to change the structure of a track while translocating along it.

Helicases move along one strand of DNA and, while doing this, should separate the opposite strand from the DNA duplex. To implement this function, they have a structure different from that of the transport motors. The helicases from the largest superfamily have two motor domains that bind and hydrolyse ATP in each operation cycle, and also other domains important for manipulating the DNA [41].

The helicase of HCV is the best-studied one, because of its important role in viral replication that makes it a major target for inhibition by drugs [42]. Single-molecule experiments with this molecular motor led to a conjecture that the inchworm ratchet translocation mechanism, described in §4, should underlie its operation [43,44]. This hypothesis could not however be checked, because mechanochemical motions were not directly experimentally accessed.

**Figure 13.**
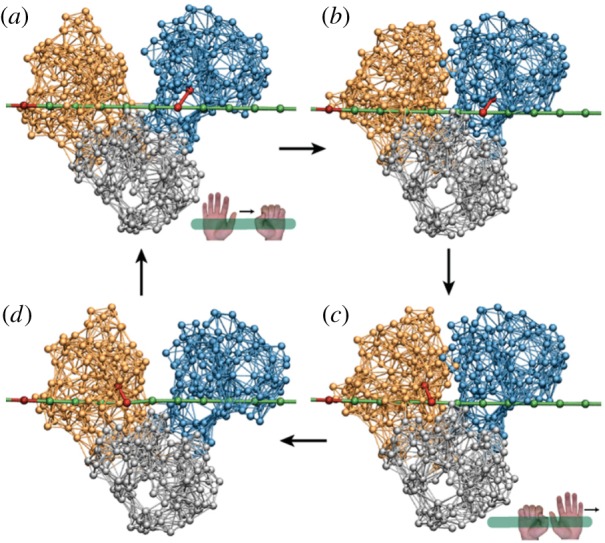
The inchworm translocation cycle of HCV helicase. Following ATP binding, the left motor domain (orange) moves towards the right motor domain (blue), so that the protein conformation is changed from (*a*) the open to (*b*) the closed one. In the closed conformation, hydrolysis occurs and its products are released (*c*), inducing the return (*d*) to the initial open conformation. Within the cycle, interactions between the motor domains and the DNA strand (green) are switched. When ATP binds, a link (red) between the right domain and the strand is established, so that this domain holds the DNA. After the hydrolysis, the reconnection occurs (*c*) and now the left domain grasps the DNA. As a result, the motor translocates itself by one DNA base in the right direction after each cycle. Adapted from [29]. See also the electronic supplementary material, video S6. (Online version in colour.)

To provide a structurally resolved description of HCV helicase operation, we have first investigated [29] the dynamics of its ligand-free elastic network. Using this coarse-grained description, the funnel conformational relaxation pattern (cf. §6) characteristic for machine proteins, ensuring robust motions of the domains, was observed. In HCV helicase, they represent opening and closing of two stiff motor domains connected through a hinge (figure 13). Remarkably, the motions were robust, but so soft that even a small perturbation localized in the region of the ATP binding pocket could produce them.

It is known that ATP binds in the open conformation to a pocket on the left domain, whereas ATP hydrolysis and product release take place in the closed conformation, when ATP comes into contact with an atomic catalytic group on the surface of the right domain. To take this into account, an ATP molecule was introduced into the model as a single bead able to bind to the pocket by forming elastic links with several beads around it. Upon binding, the links were stretched and tended to contract the pocket, inducing the closure of the motor domains. It was assumed that, in the closed state, the hydrolysis occurred and the products left the pocket. This was modelled by removing the additional ligand particle together with its links. After that the motor domains moved back to the open conformation, finishing the cycle.

Further on, it was noticed that the slow opening and closing motions along the DNA were also accompanied in the elastic network model by faster and smaller conformational changes in the DNA binding clefts of the motor domains. Consequently, the clefts could narrow or widen depending on the ligand state.

The DNA strand was modelled as a polymer chain with the beads corresponding to single DNA bases. It was assumed that, in a narrow cleft, a stiff link between a certain experimentally known residue and a DNA bead was formed, so that this motor domain could hold the DNA. When the cleft widened, this link disappeared releasing the DNA. As shown in figure 13, the opening and closing motions of the motor domains were coordinated with alternating grasping and releasing of the DNA strand in such a way that inchworm translocation could take place.

**Figure 14.**
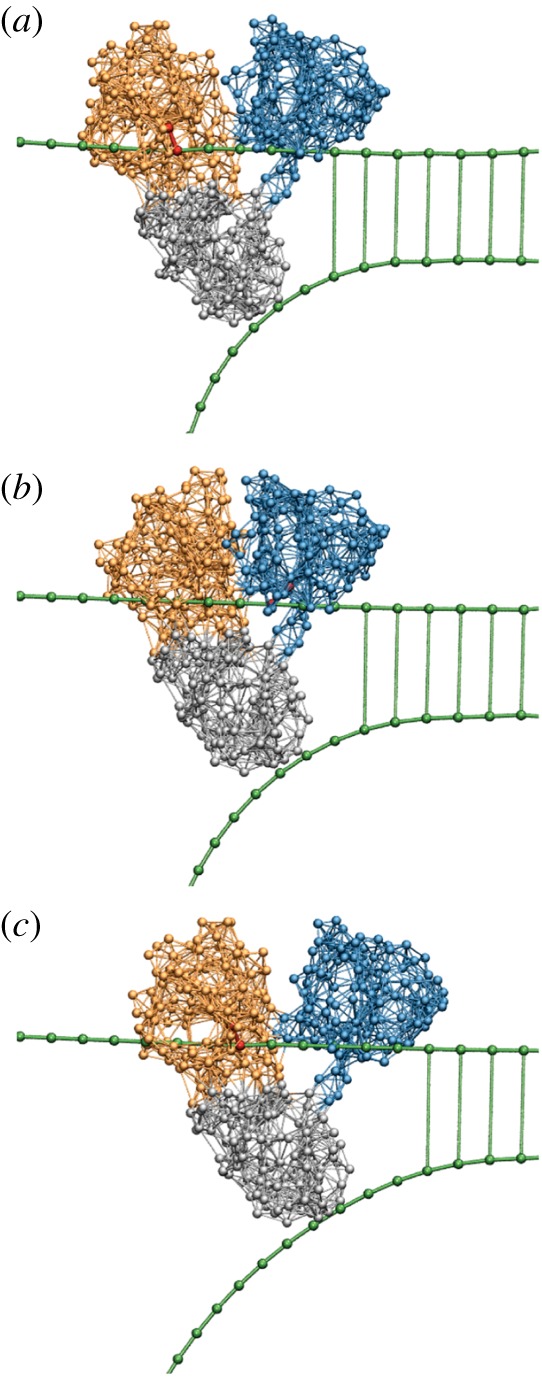
The operation of HCV helicase. Forced by translocation of the two motor domains along the upper strand, the third domain (grey) is drawn as a wedge between the two DNA strands and thus mechanically separates them. Three consequent snapshots (*a*,*b*,*c*) from a structurally resolved coarse-grained simulation [29] are displayed. See also the electronic supplementary material, video S7. (Online version in colour.)

The function of HCV helicase is to separate the two DNA strands, unzipping them. To reproduce this effect, a second polymer DNA strand was included into the model. The two strands were held together by bridge interactions between the bases in each of them. Such interactions were softening and disappeared when the distance between the strands was increased. As shown in figure 14, the translocating motor domains pushed the third passive domain into the space between the two strands. This domain thus acted as a mechanical wedge and progressively unzipped the duplex DNA.

Several operation cycles could be followed within a computer simulation [29]. The obtained results have provided a direct demonstration of the helicase operation in terms of conformational motions within the motor. They have confirmed the previously proposed inchworm operation mechanism [44]. Subsequent experimental work has allowed elucidation of this mechanism at a greater detail [45,46].

## 9. Allosteric regulation effects in proteins

Control mechanisms are required to regulate timing and speed of catalytic chemical reactions within a cell. Remarkably, such regulation can already take place at the level of a single enzyme. By binding a molecule (different from the substrate) to a specific site away from the active centre, the catalytic turnover rate of an enzyme can be modified, inhibiting or enhancing its activity. This mechanism, involving communication between different sites in a protein molecule, is known as allosteric regulation [47,48].

Allostery is important for protein machines [49]. In myosin V, for example, binding of ATP to a pocket in its head domain results in opening of the cleft where the actin filament is held. This allows the head to detach itself from the actin filament and make a step. Similar allosteric mechanisms control the operation of other cytoskeletal motors as well.

According to an intuitive interpretation of allosteric regulation, binding of a ligand should cause a local deformation around the binding centre where interactions with the ligand generate stress. This deformation propagates through the protein and, as a result, its conformation at a distant site becomes changed. Thus, functional site-to-site communication within a protein is established.

In NMR experiments, it is indeed possible [50,51] to detect communication pathways in allosteric proteins. Such pathways are formed by subsets of physically interacting residues and they link remote functional sites.

Mechanical aspects of allostery are often studied using the coarse-grained elastic network models of proteins [52,53]. In the framework of the normal-mode analysis, they can be understood as a linear response of a protein to ligand binding, modification, or release [54–57]. The effects of intramolecular communication in myosin V were moreover investigated by using the complete nonlinear elastic network model of this protein [58].

Functional intramolecular communication, underlying allosteric effects in proteins, should have emerged from the natural biological evolution of such proteins. To demonstrate this, model elastic networks with similar internal communication properties have been designed by running a computer evolution [33]. This was done similar to how the model molecular machine, described in §7, was designed—but with a different choice of the evolutionary pressure.

The optimization goal was that binding of a ligand to a pocket at one site in the network (modelled by the application of contracting forces to two beads within it) should have produced opening (or closing) of another pocket located in the remote part of the network. After each structural mutation, equations of motion for the network were numerically integrated until the final stationary state was reached. A mutation was accepted if it led to an improved allosteric response. During the evolution, the networks gradually changed their architecture, so that the desired function became implemented at the end.

Networks with either symmetric or asymmetric cooperativity could be designed. If the cooperativity is symmetric, closing of the ligand pocket leads to closing of the response pocket too. The cooperativity is asymmetric if closing of the ligand pocket results in the opening response.

**Figure 15.**
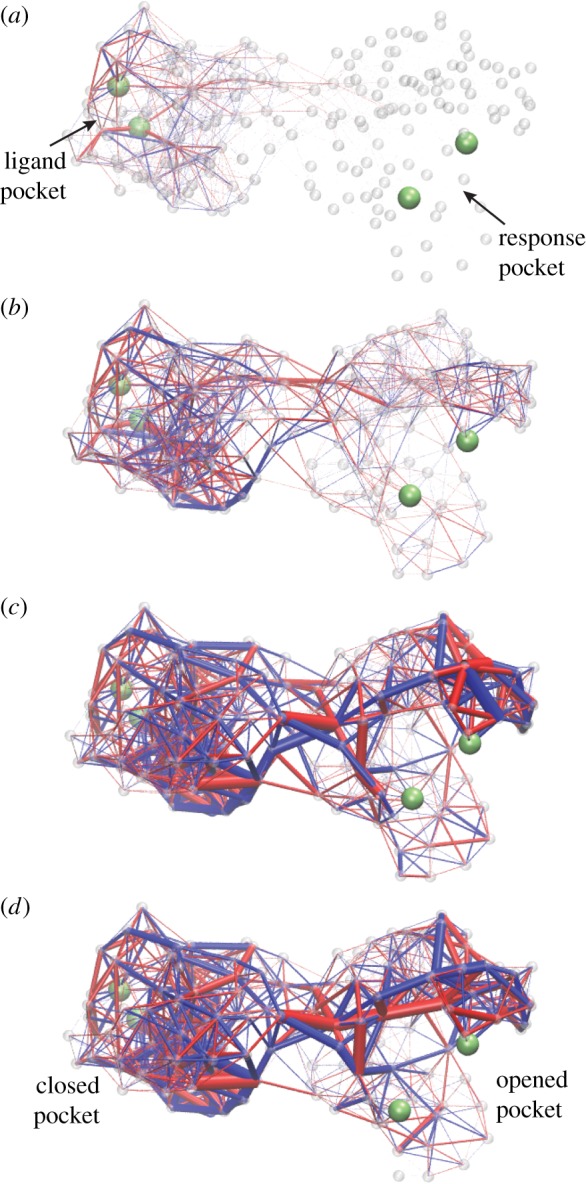
Deformation spreading through a designed elastic network with asymmetric cooperativity. Four consequent snapshots are displayed. Upon ligand binding, elastic links around the pocket in the left domain become strained (*a*). Later on, the deformation propagates into the interface between the two domains (*b*,*c*). Eventually, the links around the pocket in the right domain get strained (*c*,*d*). Thus, contraction of the ligand pocket in the left domain leads to opening of the pocket in the right domain, i.e. to an allosteric effect. Bond thickness visualizes the strain magnitude of the respective elastic link. The colour indicates whether a link is stretched (blue) or compressed (red). Contracting forces were applied to two beads (green) in the ligand pocket. The allosteric effect was quantified by measuring the distance between two beads (green) in the response pocket. Adapted from [33]. See also the electronic supplementary material, video S8. (Online version in colour.)

In a designed network, the allosteric communication results from propagation of elastic strain from the ligand binding pocket to the response site (figure 15). Here, remarkable observations can be made: out of all network links, only a minority of them gets significantly deformed and, moreover, the highly strained links form a pathway connecting the two pockets. The strain flow seems to be channelled into this pathway.

**Figure 16.**
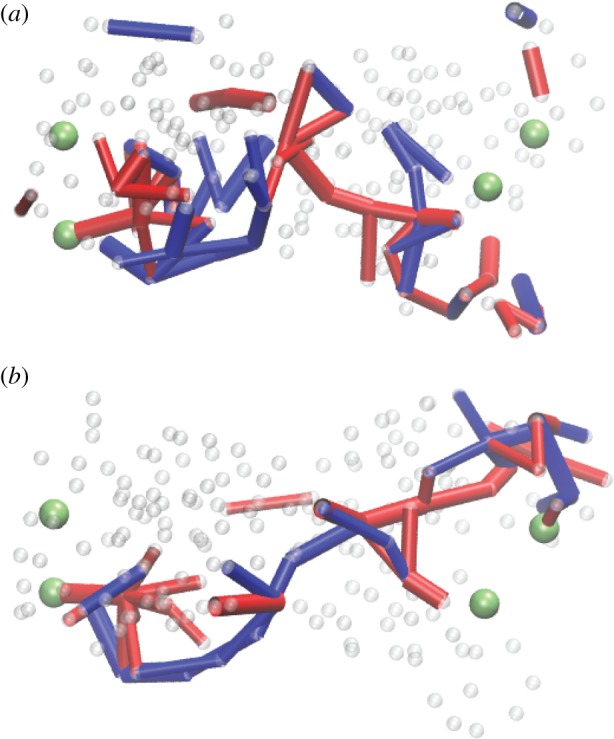
Communication pathways in designed networks with (*a*) symmetric and (*b*) asymmetric cooperativity. The network in (*b*) is the same as in figure 15. Here, only the links whose maximum absolute deformation during strain propagation has exceeded a threshold are retained. The same colour coding as in figure 15. Adapted from [33]. (Online version in colour.)

In figure 16, communication pathways in designed networks with symmetric and asymmetric cooperativity are shown. They are constructed by retaining only those links in a network whose maximum absolute deformation during the strain propagation exceeds some threshold. It can be seen that the pathways possess linear chains of nodes connecting the pockets in the two domains.

The identified pathway chains have been furthermore demonstrated to be crucial for allosteric effects. If a mutation was applied to one of the nodes along the chain, it could often disrupt the allosteric response, whereas the mutations in other nodes had only minor effects.

To verify the relevance of these evolutionary design results for real allosteric proteins, the same dynamical simulations and data analysis were repeated [33] for an elastic network of myosin V, exploring the communication between the ATP binding pocket and the actin binding cleft. The strain propagation induced by ATP binding could be observed, and it was also seen that the actin cleft became open when the deformation wave had reached it. Furthermore, the communication pathway was also determined. Remarkably, regions with accumulated strain agreed well with the known structural motifs of conserved residues that are essential for the mechanochemistry of the myosin V motor.

Hence, by running *in silico* evolution, elastic networks with the same allosteric properties as those of actual protein machines can be readily obtained. This suggests that a similar natural evolution process has led to the emergence and perfection of allosteric regulation in real proteins too. Currently, there is a considerable interest in designing artificial structures inspired by allosteric proteins—and even two-dimensional elastic grids with allosteric properties have been thus designed [59–61].

## 10. Discussion

The investigations based on elastic networks reveal that chemistry and mechanics are effectively separated in motor and machine proteins. The ‘chemistry’, i.e. the sequence of amino acids in a protein, determines into what native conformation it would fold and what would be an elastic network of that protein. Then, conformational dynamics in the folded state would be controlled by mechanical deformations and forces in this network.

From a general perspective of the theory of complex systems, low sensitivity of dynamics to chemical details can be beneficial for protein machines. If functional conformational motions in such proteins were strongly dependent on fine details, the motions could not have been robust enough to ensure reliable operation of a nano-device.

This can be especially important for virus machines, such as HCV helicase. Because viruses do not employ the proofreading and error correction machinery of the cell (e.g. [62]), every next copy of their molecular motors is typically different, without destroying in most cases the functional operation of a machine.

Studies of evolving catalytic networks as models of a living cell [63] suggest that robustness against perturbations (i.e. mutations) is intrinsically linked to the robustness with respect to environmental fluctuations. Hence, although in a different context, it is plausible that, in addition to providing robustness against mutations, separation of mechanical motions from chemical details in proteins also reduces their sensitivity to thermal fluctuations and to internal noise.

Not only functional conformational motions, but also allosteric regulation, can be already reproduced using mechanical models of elastic networks. It is remarkable that even the simplest models where differences between residues are neglected and all elastic springs are equally stiff yield reasonable predictions and are broadly employed [57,64]. By allowing residue-specific interactions and a dependence of spring constants on the natural length, the agreement with the experimental data can be nonetheless improved [21].

Computer simulations of protein dynamics based on simple elastic networks are faster than all-atom MD simulations by orders of magnitude.^3^ This dramatic difference makes it possible to statistically explore special properties of conformational relaxation in motor and machine proteins.

Structure-based coarse-grained simulations using elastic networks confirm the presence of slow ordered collective motions in such proteins. These motions are attractive, i.e. starting from different initial conditions, relaxation trajectories converge to them. Since the reduction of dynamics to a low-dimensional attractive manifold is a characteristic property of self-organization in complex systems [8], it can be concluded that self-organization at the level of a single macromolecule takes place.

The reduction to low-dimensional models with one or a few collective mechanical coordinates represents further essential simplification for protein machines. Often, but not necessarily always, collective coordinates characterize relative positions of protein domains. Biological nano-machines become then indeed similar to macroscopic mechanical devices with ordered and coordinated movements of their parts [1].

In this review, the attention was focused on simple, but still structurally resolved dynamical descriptions. It should be however stressed that, additionally, there are efficient models of molecular motors without the structural resolution. These models, out of the scope of our short review, employ phenomenological descriptions with one or several mechanical coordinates combined with stochastic transitions between discrete Markov states (e.g. [65–69]). We have also not reviewed important reduced models based on the low-energy Brownian ratchet mechanisms [70,71].

A limitation of simple elastic network models, considered in our review, is that they cannot provide for partial unfolding and refolding, i.e. for ‘cracks’ in a protein [22,72]. The topology of a network is determined by distances between residues in a chosen reference state and it is not permitted to change. Hence, plastic deformations, accompanied by breakup of existing elastic links and/or creation of new links, do not take place. Systematic incorporation of plastic deformations into such models is an important problem for further research.

It cannot be accidental that elastic networks already allow one to reproduce principal features of conformational dynamics in protein machines. However, these models remain so far phenomenological and their relationship to the underlying all-atom dynamical descriptions still has to be elucidated. On the other hand, further analytical and numerical investigations are also needed to get better general understanding of nonlinear dynamics in elastic networks.

## Supplementary Material

Video 1

## Supplementary Material

Video 2

## Supplementary Material

Video 3

## Supplementary Material

Video 4

## Supplementary Material

Video 5

## Supplementary Material

Video 6

## Supplementary Material

Video 7

## Supplementary Material

Video 8

## Endnotes

1 However, ATP synthesis becomes possible if sufficiently strong forces are applied and this is indeed taking place, for example, in F1-ATPase.

2 Similar ratchet mechanisms are however possible in rotary molecular motors.

3 Often, elastic network simulations for proteins can be already performed using desktop PCs.

## Data accessibility

This article has no additional data.
